# 

**Published:** 1885-02

**Authors:** 


					﻿FEBRTJJkRY, 1885.
OLD SERIES
Vol. xxv.
NEW SERIES? I
Vol. I.-No. 12.7 fl
1855 O”
feta
IlEDIGAl^lU^M
JOURNAL
11 W.F.WESTM0RELAND,tO .’
x H.V.M.MILLER,M.D.LL.D.^i
'	JAMES A.GRAY, M.D.^
twiBLlSHED BY JAMES P.HARRISON&.CQ^F
W	Atlanta,ga,
SUBSCRIPTION: 2.50 A YEAR.
				

